# Comparative analyses of copy number variations between *Bos taurus* and *Bos indicus*

**DOI:** 10.1186/s12864-020-07097-6

**Published:** 2020-10-01

**Authors:** Yan Hu, Han Xia, Mingxun Li, Chang Xu, Xiaowei Ye, Ruixue Su, Mai Zhang, Oyekanmi Nash, Tad S. Sonstegard, Liguo Yang, George E. Liu, Yang Zhou

**Affiliations:** 1 Key Laboratory of Agricultural Animal Genetics, Breeding and Reproduction of Ministry of Education & College of Animal Science and Technology, Huazhong Agricultural University, Wuhan, 430070 China; 2 Animal Genomics and Improvement Laboratory, BARC, USDA-ARS, Building 306, Room 111, BARC-East, Beltsville, MD 20705 USA; 3 College of Animal Science and Technology, Yangzhou University, Yangzhou, 225009 China; 4 Centre for Genomics Research and Innovation, National Biotechnology Development Agency, Abuja, Nigeria; 5 Acceligen, 3388 Mike Collins Drive, Eagan, MN 55121 USA

**Keywords:** Copy number variation (CNV), Indicine, Taurine, Lineage-differential, CNV boundaries

## Abstract

**Background:**

*Bos taurus* and *Bos indicus* are two main sub-species of cattle. However, the differential copy number variations (CNVs) between them are not yet well studied.

**Results:**

Based on the new high-quality cattle reference genome ARS-UCD1.2, we identified 13,234 non-redundant CNV regions (CNVRs) from 73 animals of 10 cattle breeds (4 *Bos taurus* and 6 *Bos indicus*), by integrating three detection strategies. While 6990 CNVRs (52.82%) were shared by *Bos taurus* and *Bos indicus*, large CNV differences were discovered between them and these differences could be used to successfully separate animals into two subspecies. We found that 2212 and 538 genes uniquely overlapped with either indicine-specific CNVRs and or taurine-specific CNVRs, respectively. Based on *F*_ST_, we detected 16 candidate lineage-differential CNV segments (top 0.1%) under selection, which overlapped with eight genes (*CTNNA1, ENSBTAG00000004415, PKN2, BMPER, PDE1C, DNAJC18, MUSK,* and *PLCXD3*). Moreover, we obtained 1.74 Mbp indicine-specific sequences, which could only be mapped on the *Bos indicus* reference genome UOA_Brahman_1. We found these sequences and their associated genes were related to heat resistance, lipid and ATP metabolic process, and muscle development under selection. We further analyzed and validated the top significant lineage-differential CNV. This CNV overlapped genes related to muscle cell differentiation, which might be generated from a retropseudogene of *CTH* but was deleted along *Bos indicus* lineage.

**Conclusions:**

This study presents a genome wide CNV comparison between *Bos taurus* and *Bos indicus*. It supplied essential genome diversity information for understanding of adaptation and phenotype differences between the *Bos taurus* and *Bos indicus* populations.

## Background

In cattle, *Bos taurus* and *Bos indicus* are two main subspecies that supply beef and milk for human daily life in the whole world. Large differences exist between them in terms of the phenotypes and geographical distributions [1]. *Bos indicus* has prominent hump and shows stronger resistances to heat, drought and diseases [2]. In addition, multiple early studies have shown that the meat characteristics were different between the two subspecies [3–5]. A number of studies have compared their genetic differences in terms of SNP (Single Nucleotide Polymorphism), indel and microsatellite on the genome-wide level [6–8]. The two sub-species have their unique alleles and QTLs (Quantitative Trait Loci), as reported by genome-wide association studies. All of these illustrated large differences between *Bos taurus* and *Bos indicus* in their genomes, and many variations were probably associated with their specific phenotypes [9].

However, their genome differences were not well understood. Especially, the studies of the large genomic structural variations just emerged recently [10–12]. Copy number variation (CNV) is a kind of large genomic structural variations, which ranges from 50 base pairs (bp) to 5 million base pairs (Mbp) [13]. Compared to the other types of genomic variants like SNPs, it shows more drastic effects on gene expression and function, such as altering gene dosage, disrupting coding sequence, or perturbing long-range gene regulation [14]. Moreover, the CNV status like total deletion in one population but not the other can help to detect the lineage-specific or lineage-differential genome sequences between two populations [15]. We previously compared CNV between the Nellore (one *Bos indicus* breed) and *Bos taurus* using the BoivneHD SNP array, and reported 1.22 Mbp lineage-specific genome sequences [15]. We further performed a population-scale CNV study using genome sequencing and CGH (Comparative Genomic Hybridization) array data based on the cattle assembly UMD3.1 [16]. Several genes that under selection between the two sub-species were found [16]. Recently, large genomic differences were detected between Angus (one *Bos taurus* breed) and Brahman (one *Bos indicus* breed) by comparing their high-quality phased genome assemblies using the trio-binning method [12]. Immune- and fat acid desaturase-related genome regions were found to be under positive selection [12].

CNV can be detected based on the CGH array, SNP array and genome sequencing data on the genome-wide level [17]. Compared to the SNP array, the genome sequencing data have much higher resolution, and can map break points down to the single base pair. Multiple strategies, such as paired end mapping (PEM), read depth (RD) and split read (SR), were used to detect CNV in the second (i.e. next) generation sequencing data [18]. However, previous studies showed high proportion of false positive when only using a single strategy [19]. Combining different strategies could greatly increase the accuracy of the CNV detection. For example, two previous CNV studies for the differences between *Bos taurus* and *Bos indicus* were performed based on the RD strategy [12, 16]. RD is the most commonly used strategy to detect CNV, but less powerful when considering the accuracy of the CNV boundaries [18]. The SR and PEM strategies can make up this disadvantage of the RD strategy [18].

In this study, we combined the advantages of the CNVnator (RD strategy) and LUMPY (SR and PEM strategies) to detect and compare CNVs in 73 animals of 10 cattle breeds based on the newly updated high-quality cattle reference genome (ARS-UCD1.2). Our study will be helpful for understanding of adaptation and phenotype differences between *Bos taurus* and *Bos indicus* on the genome-wide level.

## Results

### Genome-wide CNV detection for ten cattle breeds

**Fig. 1:**
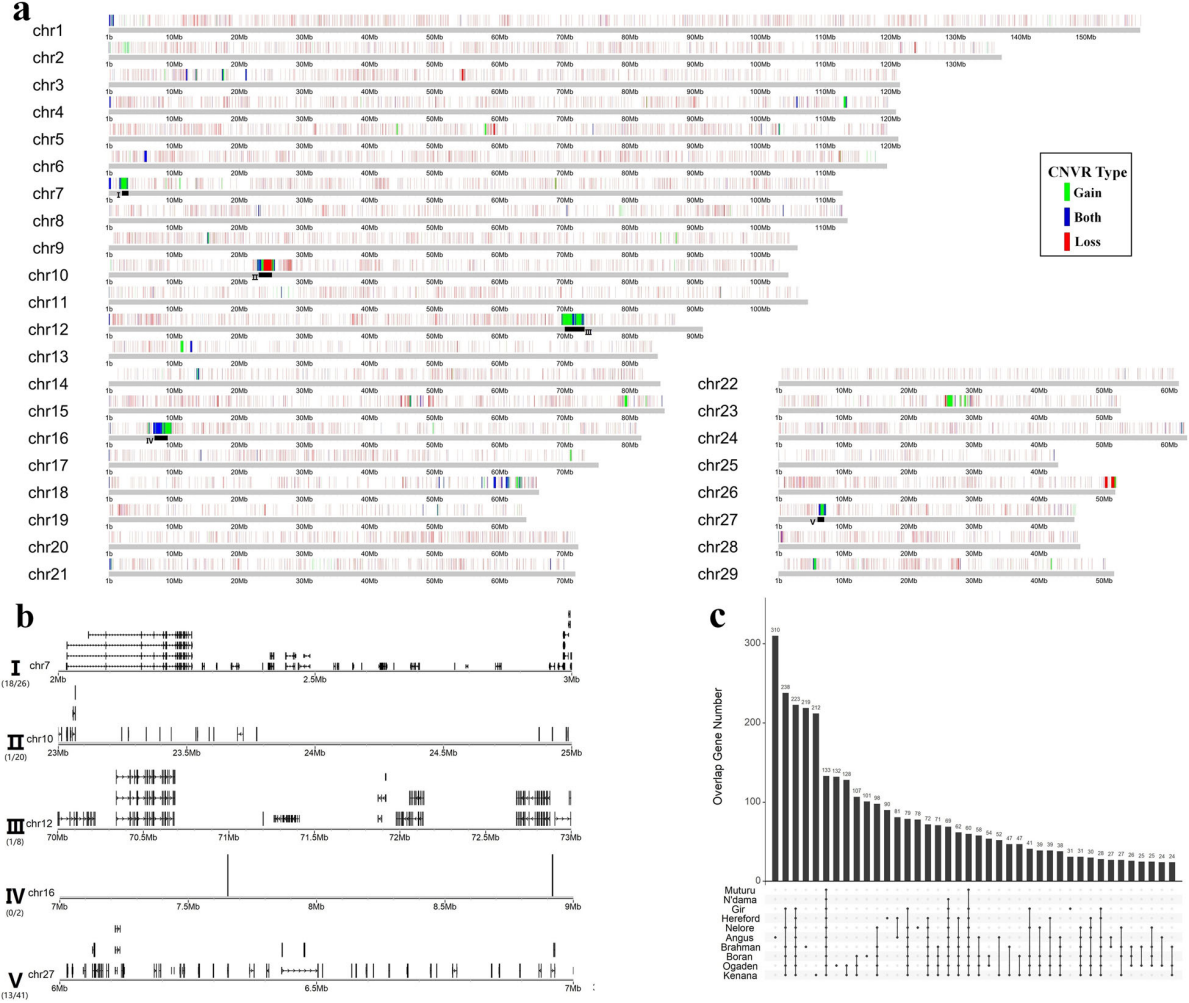
CNVR distribution in the cattle genome among different breeds. **a** CNVR distribution in the cattle genome. The black line under the CNVR represented the CNV clusters region (I-V). **b** The genes located in the CNV clusters region. **c** CNVR distribution differences among different breeds. The y axis represents the number of CNV shared by the breeds with black dots in one line

We integrated both LUMPY and CNVnator to call CNVs for 73 animals of 10 different cattle breeds using their second generation i.e. short-read sequencing data (Table 1). Totally, we retrieved 182,823 confidential CNV events for all animals, representing 66,395 distinct CNVs with an average length of 21,649 bp. These CNVs were merged into 13,234 non-redundant CNV regions (CNVRs) with a total length of ~ 40.5 Mb, corresponding to ~ 1.5% of the autosomal genome sequence (Table S1). To validate CNVRs in this study, we collected cattle CNVRs in 12 published papers and converted them to ARS-UCD1.2 coordinate using UCSC liftover tool (http://genome.ucsc.edu/cgi-bin/hgLiftOver) [15, 16, 20–29]. We found 80.7% of CNVRs detected in our study were supported by the published cattle CNVRs in length. Similar to previous studies, we obtained more deletions than duplications for all animals [16] (Fig. 1a and Table S1). We binned the cattle genome into nonoverlapping 1-Mb windows, and calculated the CNV density to search for CNV clusters in the cattle genome. We found 5 CNV clusters (9 windows) separately on the chr7, chr10, chr12, chr16, and chr27, of which over 80% in length were covered by CNVs (Fig. 1a and Table S2). Those CNV cluster regions contained 97 genes, but most of them were uncharacterized (64/97). From the characterized genes, we found those regions were enriched for gene families, such as well-known CNV-associated genes like zinc finger proteins, histones, and defensins (Fig. 1b and Table S2). When considering the distributions of these CNVR in different breeds, we found only 133 CNVRs were shared by all breeds. Most of breeds showed breed-specific CNVR distribution patterns on the genome (Fig. 1c).

**Table 1:** Samples and sequence data sets used in this study

Breeds	Subspecies	Location	Animal count	Coverage	CNV count	BioProject
Angus	*Bos taurus*	Europe	20	7–37×	34,774	PRJNA343262, PRJNA256210, PRJNA176557, PRJNA513064
Boran	*Bos indicus*	African	7	10–12×	27,996	PRJNA312138
Brahman	*Bos indicus*	Asian	4	17–19×	17,290	PRJNA432125
Gir	*Bos indicus*	Asian	3	7–15×	8787	PRJNA277147
Hereford	*Bos taurus*	Europe	12	8–17×	15,534	PRJNA343262, PRJNA176557
Kenana	*Bos indicus*	African	7	11×	29,291	PRJNA312138
Nelore	*Bos indicus*	Asian	6	6–10×	15,786	PRJNA507259, PRJNA277147
Ogaden	*Bos indicus*	African	7	10–12×	29,360	PRJNA312138
N’dama	*Bos taurus*	African	4	5–15×	1992	PRJNA604048
Muturu	*Bos taurus*	African	3	7–10×	2013	PRJNA604048

Note: The data of N’dama and Muturu were newly generated. The data for other animals were downloaded from the NCBI database

### Characterization of genes affected by CNVs in cattle

**Fig. 2:**
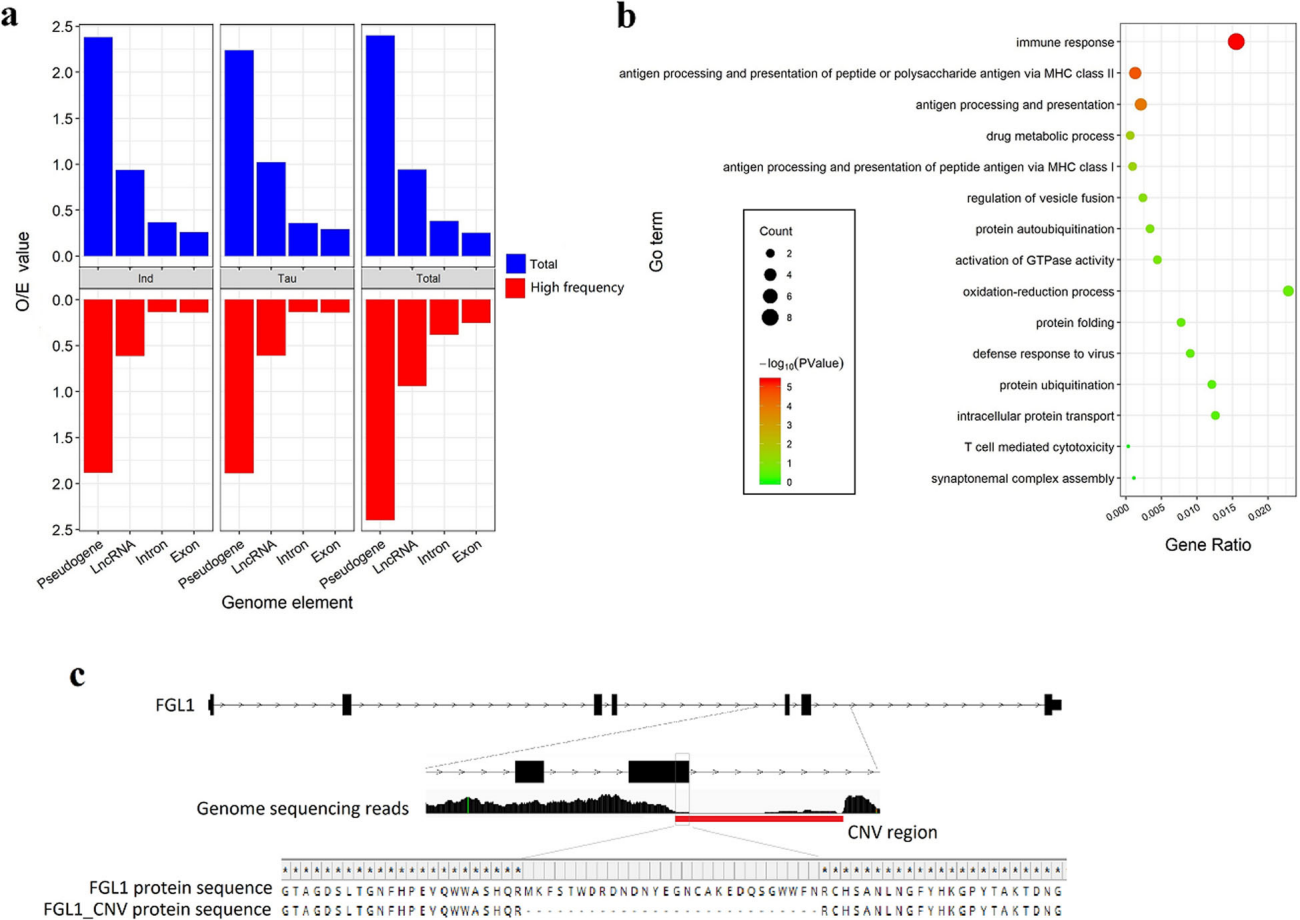
Analysis of genes affected by CNV. **a** The chance of different genome structure overlapped with the CNVR. O/E: observe/expect. **b** Gene ontology analysis for the genes with their exon overlapped with the CNVR. **c** One example of the CNV altering gene coding sequences. One CNV overlapped with a part of the sixth exon of the *FGL1* gene that caused 29 amino acid deletion. Track 1: gene structure of the cattle *FGL1* gene; Track 2: IGV result of mapped reads on the cattle genome; Track 3: the amino acid sequences of the wild FGL1 protein and the FGL1 protein with a partial deletion

We evaluated the CNVR distribution patterns in different genomic structures. In line with the previous results, the CNVR was more preferably overlapped with the pseudogenes than the transcript regions (LncRNAs and introns in the coding genes), and the coding regions (exons) had the least chance to overlap with the CNVR [30] (Fig. 2a). Totally, there were 4831 genes overlapped with CNVRs in all animals (Table S1). Among them, we found 82 genes with their exons affected by CNVR (Table S3). GO (Gene Ontology) analysis revealed that those genes were highly enriched in immune-related GO terms, such as the immune response, antigen processing and presentation of peptide or polysaccharide via HMC class II, antigen processing and presentation (Fig. 2b). When a gene’s exons overlapped with a CNV, its coding region could be seriously changed and may function differently. For example, the *FGL1* gene, overlapped by a CNV that caused 29 amino acid deletion, may produce different transcripts in different animals (Fig. 2c). To detect the effects of the high variable CNVR on the coding regions on the population level, we first merged all distinct CNVs, then dissected them to CNV segments as described previously [15]. Briefly, we first dissected CNVRs into CNV segments according to the boundaries of individual CNV calls, and then calculated the frequency of each CNV segment. Eventually, we detected 15 genes (0.31% of all genes affected by CNVs) with their exons overlapped with high frequency (≥50%) CNV segments (Table S4).

### Population genetic analysis using CNV for ten cattle breeds

**Fig. 3:**
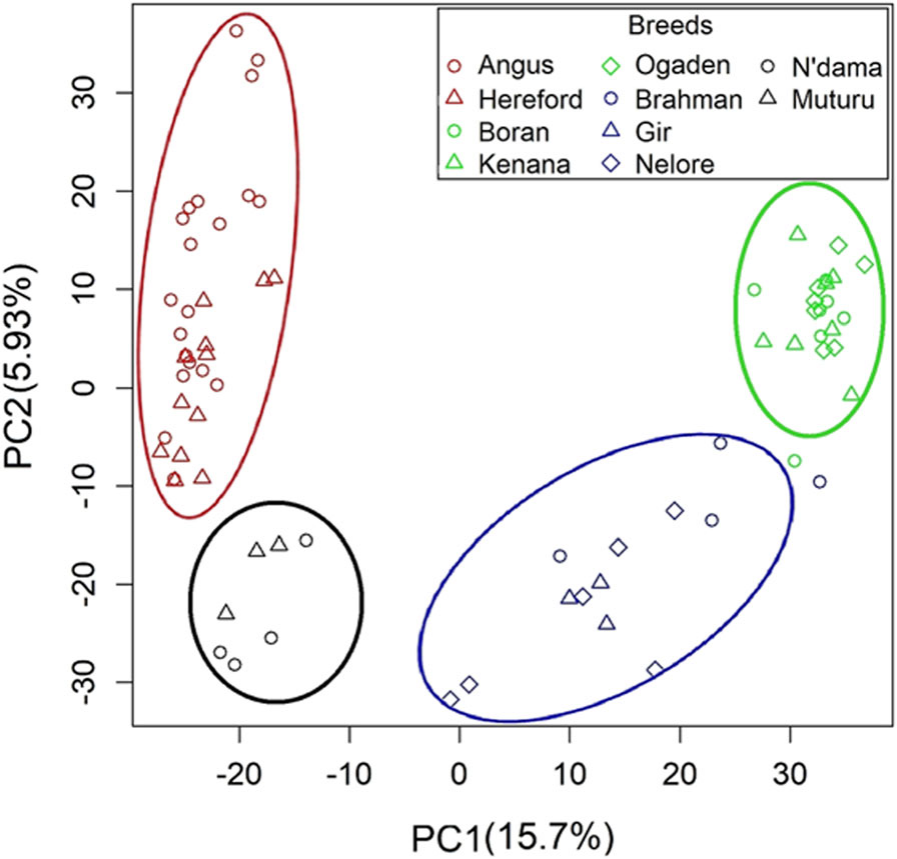
PCA analysis of the ten cattle breeds based on the CNV

To obtain the population structure of different cattle breeds based on CNV, we performed cluster, PCA (principal components analysis) and admixture analyses [31]. The CNV segment was genotyped to five types (0, 1, 2, 3, ≥4) according to its original copy number for these analyses [15]. The cluster result indicated, when consideringglobally, animals were generally separated to two large groups (*Bos taurus* and *Bos indicus*) [32]. These two branches can be divided into four subgroups (Figure S1a): Europe *Bos taurus* (Angus and Hereford), African *Bos taurus* (N’dama and Muturu), Asian *Bos indicus* (Brahman, Gir and Nelore), African *Bos indicus* (Boran, Kenana and Ogadan) [33]. This was supported by the PCA result that the PC1 was successfully divided the samples of *Bos taurus* from those of *Bos indicus* (Fig. 3a). In the admixture analysis, varying the number of presumed ancestral populations (K) recapitulated the extent of genetic divergences across breeds (Figure S1b). At K = 2, the *Bos taurus* were separated with the *Bos indicus*. At K = 3, the Asian *Bos indicus* showed a clear separation from the other groups. At K = 4, the *Bos taurus* were separated to Europe *Bos taurus* and African *Bos taurus*.

### Differential CNV segments between *Bos taurus* and *Bos indicus*

It is of note that the percentage of deletions was higher in *Bos indicus* than that in *Bos taurus* (Figure S2). This is likely related to the genome reference bias, and could reveal the existence of the sub-species-specific sequences for *Bos indicus*. We isolated unmapped reads for the *Bos indicus* cattle and successfully re-mapped them on the reference genome of the *Bos indicus* (UOA_Brahman_1) [12]. After merging, we detected 1.74 Mbp indicine-specific sequences (over 500 bp in length with at least 2 reads in coverage). The top genes in the indicine-specific sequences were involved in the regulation of Rho protein signal transduction, but their enrichment was not significant.

We compared the CNVRs between *Bos taurus* and *Bos indics*. Large differences were found between them in terms of the CNVR distribution and status. Only 6990 CNVRs (52.82%) were shared by both sub-species. *Bos indicus* contained more CNVRs (both number and length) per animal as compared to *Bos taurus* (Figure S3). We detected 2619 and 4293 genes that uniquely overlapped with CNVRs of either *Bos taurus* or *Bos indicus*, respectively (Figure S4a). The commonly overlapped genes were significantly (FDR < 0.05) enriched in the intracellular signal transduction (Figure S4b). We did not find any significantly enriched GO term (FDR < 0.05) for the genes overlapped with the taurine-specific CNVRs. However, we found that the genes overlapped with *Bos indicus*-specific CNVRs were significantly (FDR < 0.05) enriched in the regulation of Rho protein signal transduction (Figure S4b).

**Fig. 4:**
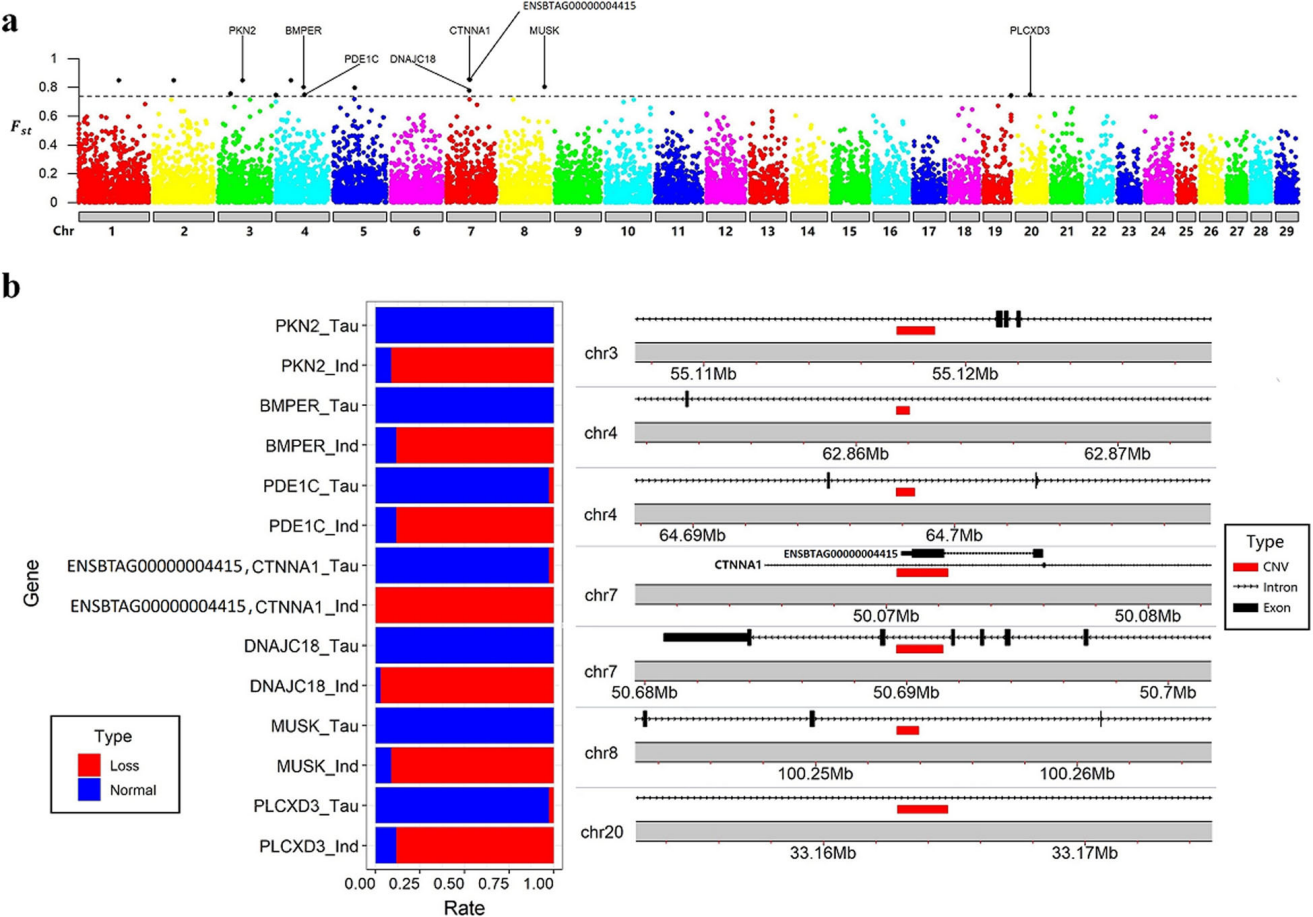
Comparisons of CNV segments between *Bos taurus* and *Bos indicus.* **a** *F*_ST_ between *Bos taurus* and *Bos indicus* at CNV segment level. The dotted line represented the top 0.1%. **b** The rate of the CNV segment status (loss and normal, no gain was found for these CNV segments) in *Bos taurus* and *Bos indicus*, and the position of differential CNV segments overlapped with genes

To fine map regions under genome selection, we applied a statistics comparison of CNV segments between *Bos taurus* and *Bos indicus* at a global level, using F-statistics. We obtained 159 most divergent CNV segments, by using the top 1% threshold (Fig. 4a and Table S5). We did not find any significant GO term for the genes overlapped with the differential CNV segments (FDR < 0.05). When we used a stricter threshold (top 0.1%), we found 16 differential CNV segments and 7 of them were overlapped with 8 different genes (Fig. 4a). The functions of those genes were dispersed in the heat stress (*DNAJC18* [34]), lipid and ATP metabolic process (*PLCXD3* [35]: GO:0006629, lipid metabolic process; *MUSK* [36]: GO:0005524, ATP binding; *PKN2* [37]: GO:0005524, ATP binding;) and muscle development (*CTNNA1* [38, 39]: GO:0051149, positive regulation of muscle cell differentiation; MUSK [40]: GO:0071340, skeletal muscle acetylcholine-gated channel clustering; PKN2 [41]). It is of note that all significant CNV segments showed high ratio of deletion in *Bos indicus*, while no change or normal in *Bos taurus* (Fig. 4b), suggesting that they are likely to be specific sequences of the *Bos taurus*.

### Possible regulation mechanism and origin of the top differential CNV

**Fig. 5:**
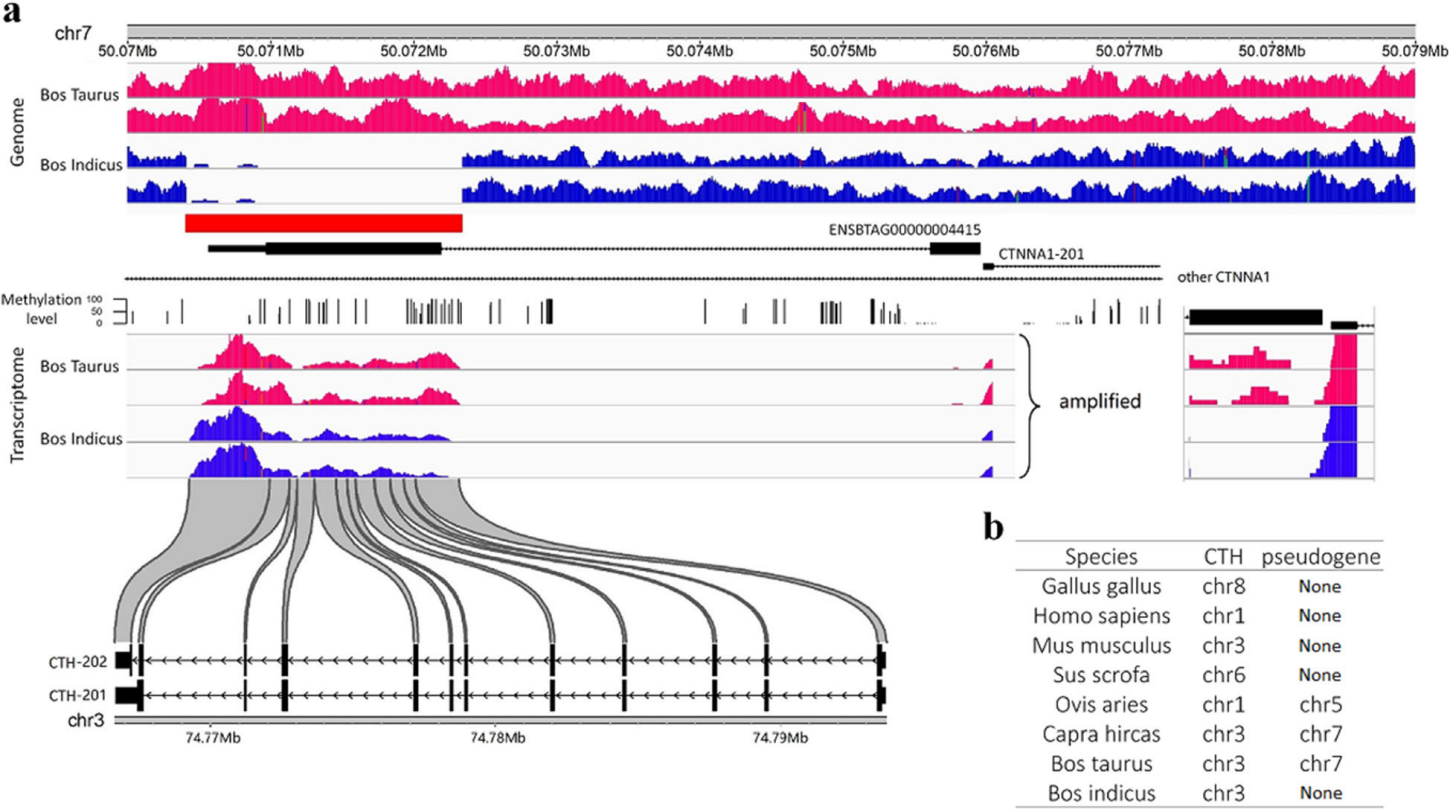
Analysis of the effects of top differential CNV segment on genes and its possible formation history. **a** Distribution of the genome sequencing and RNA sequencing reads around the CNV and the affected two genes. **b** The chromosome location of *CTH* and pseudogene *CTH* in different species

Interestingly, the top significantly differential CNV segment (chr7:50070412–50,072,341) was not only covered the second exon of the *ENSBTAG00000004415* gene (uncharacterized gene), but also located in the intron region of the *CTNNA1* gene at the same time (Fig. 4b). The *CTNNA1* expressed multiple alternative transcripts. One of the *CTNNA1* transcripts has its first exon 3 bp away from the first exon of the *ENSBTAG00000004415*. By integrating the methylation data, we showed that the two genes’ first exons were located in one HMR (hypomethylated region) with the characteristics of transcript start site (Fig. 5a). This implied that the two genes might be regulated by the methylation status of one same HMR and possibly co-expressed in different tissues with similar functions. We did blast the ENSBTAG00000004415 sequence against the cattle genome (ARS-UCD1.2) and found that the second exon of the ENSBTAG00000004415 was actually a retropseudogene of *CTH* in *Bos taurus*. Previous studies showed that both the CTH and the CTNNA1 functioned in the muscle cell differentiation [39, 42]. We speculated that this CNV segment (chr7:50070412–50,072,341) may be related to the muscle development difference between *Bos taurus* and *Bos indicus*, through regulating ENSBTAG00000004415 and CTNNA1.

To validate this differential CNV segment, we first visualized the mapped reads on the reference genome and received a consistent result with the CNV status for all animals used in this study (Fig. 5a). Next, we used the PCR to check the existence of this CNV segment in 22 *Bos taurus* (6 Holstein, 4 Jersey, 6 Angus, 6 Hereford) and 19 *Bos indicus* (6 Nelore, 3 N’dama, 4 Muturu, 6 Brahman). The result showed that all *Bos indicus* animals were deletion, while all *Bos taurus* animals were normal with 2 copies, which confirmed our observation in the genome sequencing analysis. We further checked the reads mapped on the ENSBTAG00000004415 using the RNA sequencing data for *Bos taurus* and *Bos indicus*. Although we could not clearly distinguish the reads on the second exon that were transcribed from *CTH* or *ENSBTAG00000004415*, we observed few reads mapped on the first exon in *Bos Taurus*, but not in *Bos indicus* (Fig. 5a). This implied that *ENSBTAG00000004415* might not be expressed in *Bos indicus*, possibly due to the deletion of the second exon.

We did a preliminary check of the existence of the *CTH* retropseudogene in the species with high-quality reference genomes to confirm the formation history of the CNV during evolution. We found that the *CTH* retropseudogene also appeared in the other ruminant animals, such as goat and sheep, but not in the non-ruminant animals like human, pig and chicken (Fig. 5b). Combined with the specific deletion in the *Bos indicus*, we speculated that the *CTH* mRNA insertion might happened before the ruminant speciation but lost in the *Bos indicus* lineage.

## Discussion

To date, most studies used the RD strategy to detect CNV, which is fast and easy to obtain the exact copy number of the CNV [43]. But in the livestock study, the sequencing depth is usually limited by the current funding, which will affect the RD strategy to obtain high confident CNVs and high accurate CNV boundaries [43]. This will seriously affect further analyses, like overlapping results with genes, promoters, enhancers and other functional genome structures. Especially in the time of omic data, the false positive will be easier amplified to reach wrong conclusions [44]. In this study, we integrated the RD strategy with the RP and SR strategies, which are based on orientations and distances between the paired reads and the read split events, respectively. They do not request high read numbers or read depths, but instead 2 or 3 read pairs are usually enough [18]. This will help to decrease the false positive rate of CNV detection, as compared to the single strategy.

We confirmed that CNV has the least chance to appear in the exon region that is consistent with the common perception. This supplied evidence that the CNV has more drastic effects on gene expression and function [14]. Especially when disrupting coding sequence, the harmful or lethal CNVs will have more chances to be selectively eliminated. Here, we also found the genes with the exon overlapped with the CNV were highly enriched in the immune function. This is supported by dozens of research results that the immune gene was highly diverse and complexity among individuals [45–47]. In the cattle genome, chr23 and chr15 have drawn attention of the CNV studies, because of their enriched major histocompatibility complex (MHC) genes and olfactory receptor (OR) genes. We found 5 other regions in different chromosomes that were enriched CNVs in the cattle genome. This may be also caused by the high variable gene families among different animals, such as ZNF and beta-defensins [48, 49].

In our study, we selected samples of cattle representing four regions: Europe *Bos taurus*, African *Bos taurus*, Asian *Bos indicus*, and African *Bos indicus*. Our classification and evolution results using the CNV segment were mostly supported by the previous studies using the SNP [32, 50]. African *Bos indicus* exhibited high levels of shared genetic variation with Asian *Bos indicus* but not with African *Bos taurus*, probably because of their recent divergence [33]. Overall, our population analyses successfully divided the animals into *Bos taurus* and *Bos indicus*. This supplied confidence to do a further genome comparison analyses at the CNV level. Additionally,, we further overcame the current problems for the CNV population study, namely complexity for genotyping and inconsistent boundary mapping for different individuals.

We found 1.74 Mbp indicine-specific sequence that could only be mapped on the Brahma (*Bos indicus*) reference genome. Interestingly, the function of genes in these regions were similar to the genes in *Bos indicus*-specific CNVRs that were enriched in the regulation of Rho protein signal transduction. The Rho is an RNA-binding protein with the capacity to hydrolyze ATP. Previous studies proved that it plays important roles in the heat stress, which was exactly in line with the heat resistance characteristics of *Bos indicus* [51]. Using F-statistics, we found 16 lineage-differential CNV segments between *Bos indicus* and *Bos taurus*. Compared to the previous studies [12, 16], 8 genes overlapped by the lineage-differential CNVs were novel. However, both of our and previous studies showed similar functions (heat resistance, lipid and ATP metabolic process) for the differential CNV between *Bos taurus* and *Bos indicus* [12, 16]. We also found genes for muscle development might be under selection, which would provide genetic evidences for the meat difference between the two cattle subspecies.

We explored the origin for the top lineage-differential CNV between *Bos taurus* and *Bos indicus*. We found this CNV was a retropseudogene of the *CTH*. The retrocopy of one gene could insert in a non-functional sequence, disrupt a gene or form a fusion transcript with unpredictable consequences [52]. A previous study reported that the HIV resistance of owl monkeys caused by the insertion of a Cyclophilin A cDNA into the TRIM5 gene [53]. In our study, we found the insertion of the retropseudogene of *CTH*, together with one extra exon, formed the *ENSBTAG00000004415* gene that might have function in the muscle development. This retro event might have happened before the speciation of ruminant, as it only appeared in the ruminant. However, it was totally deleted in the *Bos indicus*. We speculate that it might be selectively erased during or after the separation of *Bos indicus* from *Bos taurus* to better adapt to their environments.

## Conclusions

Based on the new high-quality cattle reference genome ARS-UCD1.2, we detected 66,395 distinct CNVs (~ 1.5% of the reference genome in length) in 73 animals of 10 different cattle breeds, through taking advantages of different CNV calling strategies. Large CNV differences were found between *Bos taurus* and *Bos indicus*. We obtained 1.74 Mbp indicine-specific sequence that could only be mapped on the Brahma (*Bos indicus*) reference genome, and 16 lineage-differential CNV segments between the two sub-species. Further functional analyses showed that genes related to heat resistance, lipid and ATP metabolic process, muscle development were possibly under selection. We successfully validated the top significant lineage-differential CNV, which might be generated from a retropseudogene of CTH but was deleted along *Bos indicus* lineage. Our study supplied essential information to promote the understanding of adaptation and phenotype differences between *Bos taurus* and *Bos indicus* at the CNV level

## Methods

### Data generation and collection

In this study, we totally collected whole genome sequencing data of 73 animals. Among them, 66 genome data of 8 cattle breeds were downloaded from the NCBI database, including 20 samples of Angus, 12 samples of Hereford, 7 samples of Boran, 4 samples of Brahman, 3 samples of Gir, 7 samples of Kenana, 6 samples of Nelore, 7 samples of Ogaden. We sampled another 4 N’dama and 3 Muturu from African *Bos taurus*. DNA was extracted from ear-tissue samples that were obtained from animals in farms of the Institute of Agricultural Research and Training, Ibadan. The animals were released immediately after collecting the samples. Paired-end libraries were constructed and sequenced using the Hiseq 2000 platform (Accession number for GEO database: PRJNA604048). In addition, to analyze the differential CNVs between the two subspecies, we downloaded four liver RNA sequencing data of *Bos taurus* (SRR1607562 and SRR1607566) and *Bos indicus* (SRR6798334 and SRR6798339), one whole genome bisulfate sequencing data (SRX3367857) of the blood genome DNA from the NCBI database. The detail information of all the data used in this study can be found in the supplement data (Table S6).

### Identification of cattle CNV

The adapter and low-quality reads were filtered using the NGS QC Toolkit (v2.3.3) software using the parameters as -p 8 -l 70 -s 20 -z g. The clean reads were mapped on the latest cattle genome reference (ARS-UCD1.2, a Hereford-based genome assembly) using the BWA (v 0.7.17) software with the default parameters and the MEM algorithm. We called CNV by combining the advantages of both LUMPY and CNVnator. Briefly, the sorted bam files were first processed using PEM and SR strategies in LUMPY (−mw 4 -tt 0 -pe) to obtain the type and the accurate CNV boundaries at a single base resolution. Then we applied a RD strategy using CNVnator software with a bin size of 350 bp to annotate the detailed copy number [54]. Only CNVs identified as the same type in both LUMY and CNVnator were considered as confidential CNV for further analysis in this study. The CNVR were generated by merging the overlapped CNVs from different individuals. After considering the intersections between results of LUMPY and CNVnator, only CNVR supported by at least 4 animals were kept (Table S1). This illustrated the high confidence for CNVRs we detected.

### Differential CNV segment identification

CNVs were divided to segments using a python script according to the different boundaries, as described before [55]. We defined the genotype of CNV segments for each individual according to the type of unique CNV it belonged to. In order to be consistent, each produced CNV segment would have same boundaries in different individuals. Only CNV segment longer than 50 bp was considered for further genotyping. The F-statistics (*F*_*ST*_) value was calculated according to the formulation in previous study [56, 57]. *F*_*ST*_ = (Ht − Hs) / Ht; Ht = 1 − Pti^2^; ti = ((xi · Nx) + (yi · Ny)) / (Nx + Ny); Hs = ((1 − Pxi^2^) · Nx + (1 − Pyi^2^) · Ny) / (Nx + Ny), where xi and yi are the population frequencies of allelic CNV segment number i (i = A0, A1, A2, A3, A4 or > A4) in population X and Y, respectively, Nx and Ny denote the number of individuals in population X and Y, and ti is a weighted average of xi and yi.

### CNV annotation and gene functional enrichment analysis

The annotation files used in this study were downloaded from the Ensembl database (http://asia.ensembl.org/index.html). The overlap cases were detected using R script (v3.6.0) and defined as at least one bp overlap. The gene IDs were used to query gene ontology terms using DAVID with Fisher’s exact test (https://david.ncifcrf.gov/).

### RNA sequencing data and WGBS data processing

We used the HISAT2 to map the read from RNA sequencing data on the cattle reference genome using the default parameters. The clean reads for RNA sequencing data were aligned on the reference genome (ARS-UCD1.2) along with annotated genes in the Ensembl database (http://asia.ensembl.org/index.html) using the HISAT2 v2.1.0 with the default parameters to generate the bam file containing the mapping information. The .bam file was inputted to the IGV (Integrative Genomics Viewer) software (http://software.broadinstitute.org/software/igv/) to visualize the reads distribution in specific regions. The WGBS data was processed according to our previous study [58]. In brief, programs FastQC (v 0.11.2) and Trim Galore (v 0.4.0) were used to generate sequence quality reports and to trim low-quality bases and the adapter sequences, respectively. High-quality reads were aligned to the reference genome (ARS-UCD1.2) using bowtie2 under the Bismark software (0.14.5) with the parameters -p 3 -N 1 -D 20. The methylcytosine information was extracted using the bismark methylation extractor, after removing the duplication reads. The first 6 bp were ignored for the paired-end reads to decrease the potential effects of severe bias toward nonmethylation in the end-of-reads caused by end repairing.

### Validation of differential CNV segment using PCR

Genomic DNA for different breeds extracted from semen were obtained from the Meat Animal Research Center (MARC) beef cattle diversity panel, version 2.1; MBCDP2.1, as described before (https://pubmed.ncbi.nlm.nih.gov/11252171/). We designed primers (forward: CTGAGCAACTGCCATTCCGT; reverse: ACCATGACAAGCTTACTAGGGGT) based on the newest version of the cattle genome (ARS-UCD1.2). The PCR amplification was performed with 50 μL reaction volume according to Taq DNA polymerase manufacturer’s protocol (Taq PCR Master Mix Kit, Qiagen, Hilden, Germany), and the genomic DNA was amplified on a bioRad MyIQ thermocycler. The touchdown PCR cycle for target region amplification was as follows: initial denaturation at 94 °C for 4 min; followed by 18 cycles of 94 °C for 30s, annealing at 68 °C ~ 50 °C (decrease 1 °C per cycle) for 30s; 22 cycles of 94 °C for 30s, annealing at 50 °C for 30s; primer extension at 72 °C for 1 min; final extension at 72 °C for 10 min. All the amplified products were run in 1.5% agarose gel.

## Supplementary information

**Additional file 1: Figure S1** Population analysis of the ten cattle breeds using CNV segments. a: Cluster analysis of the ten cattle breeds using CNV segments; b: Admixture analysis of the ten cattle breeds using CNV segments.

**Additional file 2: Figure S2** Rate of CNV in type of deletion (number) for each cattle breeds.

**Additional file 3: Figure S3** Comparisons of CNV between *Bos taurus* and *Bos indicus.*

**Additional file 4: Figure S4** Analyses of genes overlapped with CNVRs in *Bos taurus* and *Bos indicus.* a: Venn plot for number of genes overlapped with CNVRs; b: Gene ontology analyses for the genes overlapped with CNVRs in *Bos taurus* and *Bos indicus.*

**Additional file 5: Table S1** Information of the CNVR in the ten cattle breeds. **Table S2.** Information of the CNV cluster in the cattle genome of the ten cattle breeds. **Table S3.** Information of the genes with their exon overlapped with CNVRs. **Table S4.** Information of the genes overlapped with high frequency (> = 50%) CNV segments. **Table S5.** F-statistics comparison result between *Bos Taurus* and *Bos indicus* based on CNV segments (top 1%). **Table S6.** Genome data information for the CNV detection.

## Data Availability

The new generated whole genome sequencing raw data were submitted to the NCBI GEO database (https://www.ncbi.nlm.nih.gov/gds/?term=) with accession number: PRJNA604048. The 66 downloaded whole genome sequencing data of 8 cattle breeds were from the NCBI database (https://www.ncbi.nlm.nih.gov/sra/) under the BioProject with accession numbers in Table 1. The accession ID for each dataset can be found in Table S6. The four liver RNA sequencing data of *Bos taurus* and *Bos indicus* can be acquired with SRR1607562, SRR1607566, SRR6798334 and SRR6798339, and the one whole genome bisulfate sequencing data of the blood genome DNA can be acquired with SRX3367857 from the NCBI database (https://www.ncbi.nlm.nih.gov/sra/).

## Abbreviations

CNVs: Copy number variations
SNP: Single nucleotide polymorphism
GWAS: Genome-Wide Association Studies
PEM: Paired end mapping,
RD: Read depth
SR: Split read
