# Supplementary figures and images for: Comparative analyses of copy number variations between Bos taurus and Bos indicus
Source: BMC Genomics. 2020 Oct 1;21:682. doi: 10.1186/s12864-020-07097-6 (PMC7528262; doi:10.1186/s12864-020-07097-6)

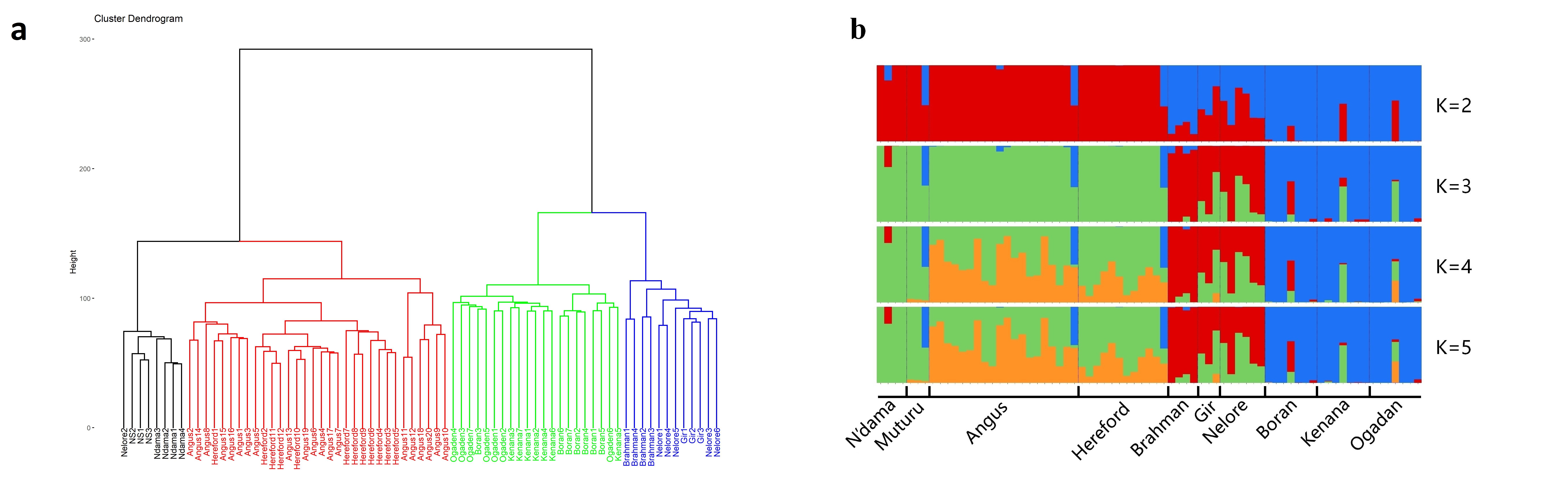

Supplement: Supplementary file 1 — Additional file 1: Figure S1 Population analysis of the ten cattle breeds using CNV segments. a: Cluster analysis of the ten cattle breeds using CNV segments; b: Admixture analysis of the ten cattle breeds using CNV segments. [file 12864_2020_7097_MOESM1_ESM.jpg]

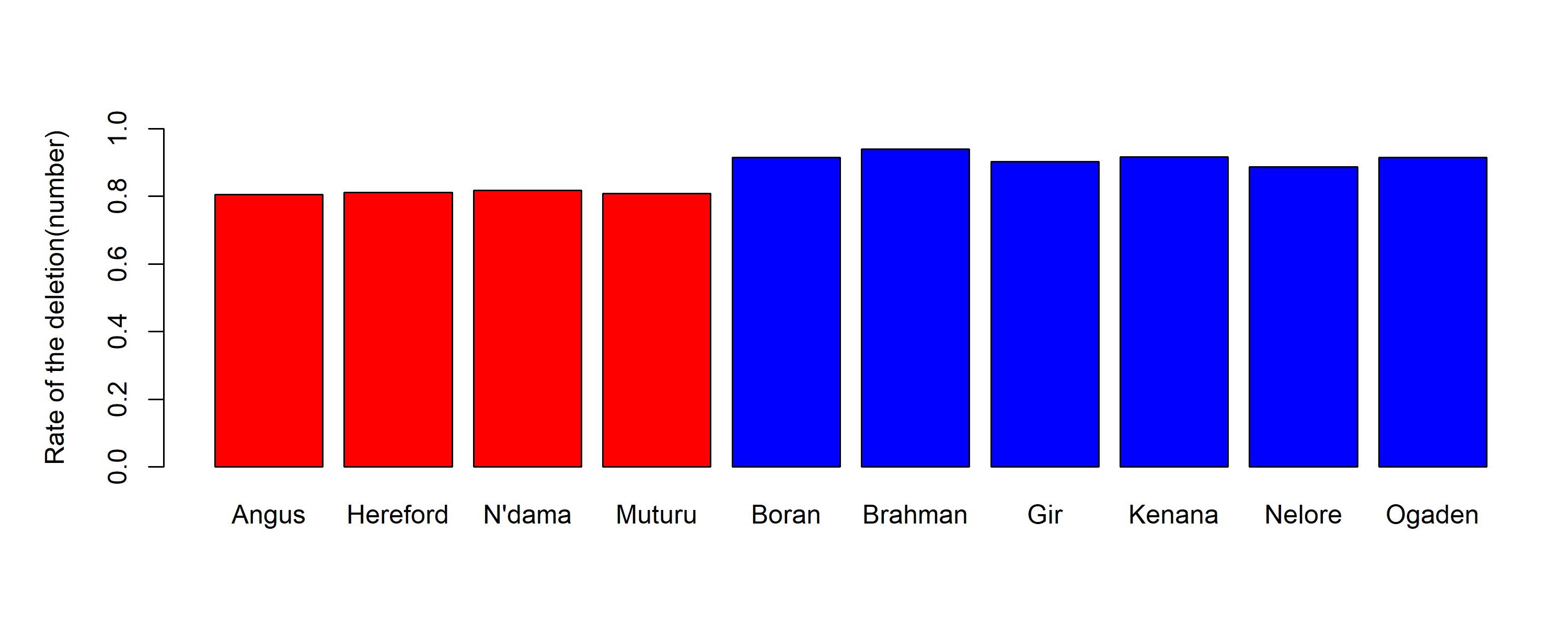

Supplement: Supplementary file 2 — Additional file 2: Figure S2 Rate of CNV in type of deletion (number) for each cattle breeds. [file 12864_2020_7097_MOESM2_ESM.jpg]

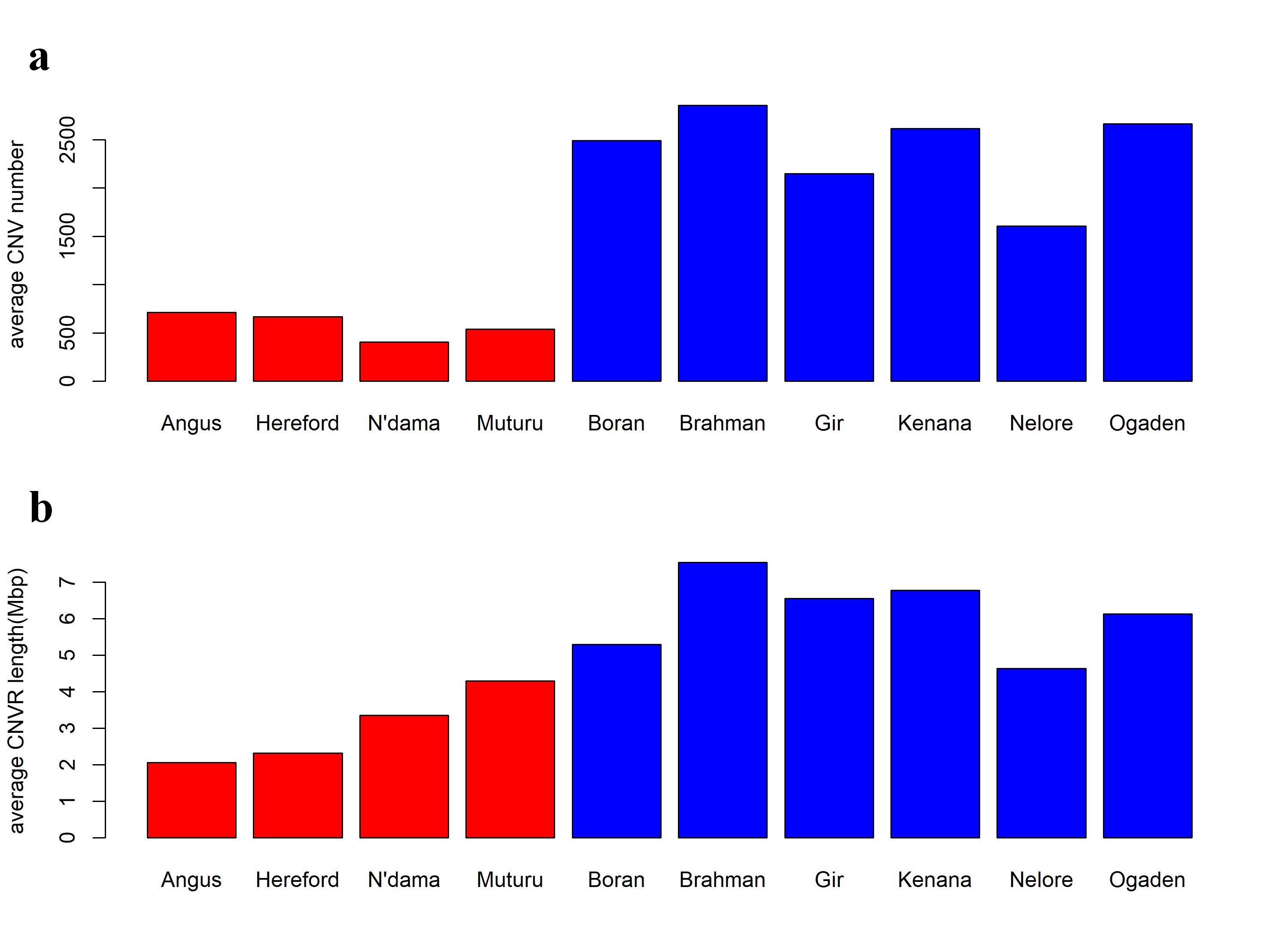

Supplement: Supplementary file 3 — Additional file 3: Figure S3 Comparisons of CNV between Bos taurus and Bos indicus. [file 12864_2020_7097_MOESM3_ESM.jpg]

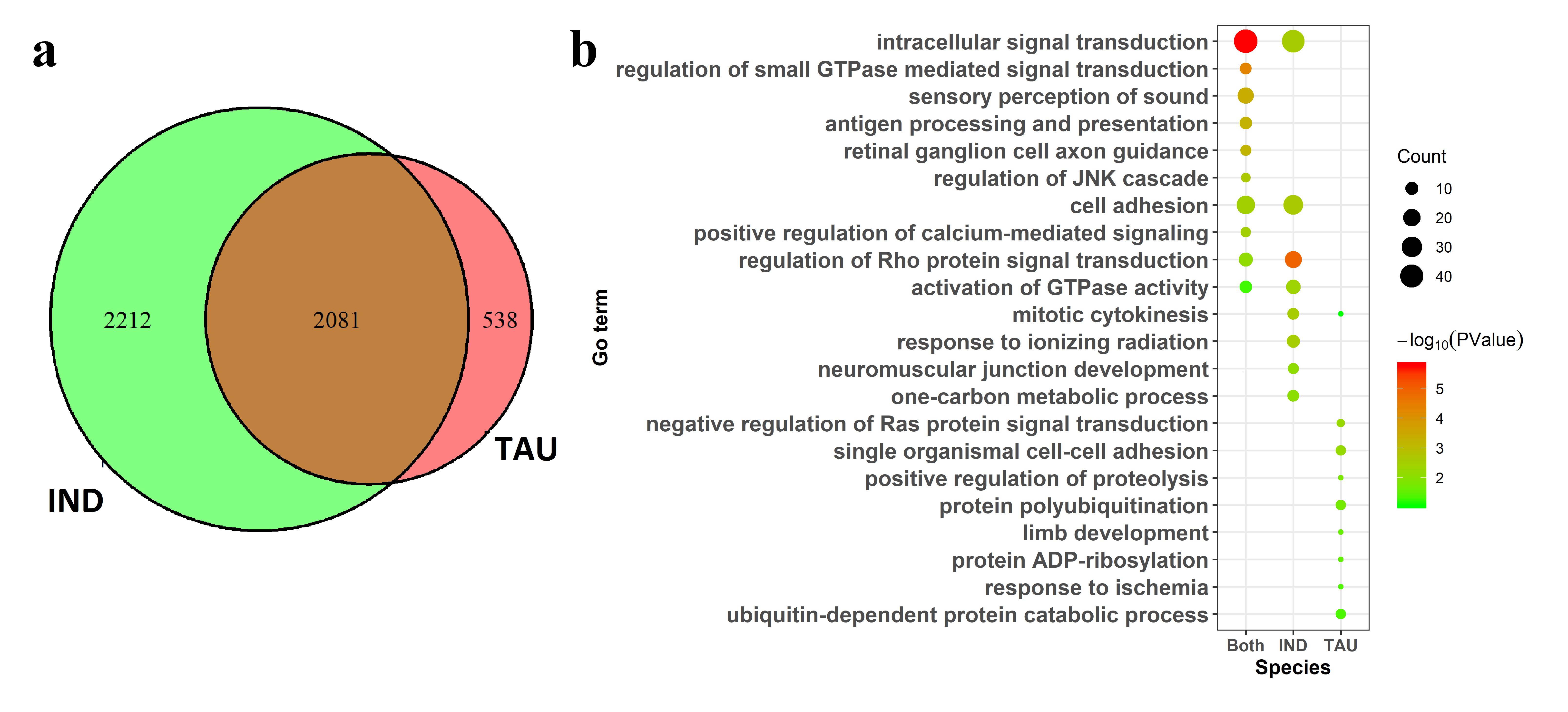

Supplement: Supplementary file 4 — Additional file 4: Figure S4 Analyses of genes overlapped with CNVRs in Bos taurus and Bos indicus. a: Venn plot for number of genes overlapped with CNVRs; b: Gene ontology analyses for the genes overlapped with CNVRs in Bos taurus and Bos indicus. [file 12864_2020_7097_MOESM4_ESM.jpg]
